# Public Health in Antarctica

**DOI:** 10.7759/cureus.50263

**Published:** 2023-12-10

**Authors:** Ravindra Nath, Pooja Sindwani

**Affiliations:** 1 Preventive Medicine, National Centre for Polar and Ocean Research, Ministry of Earth Sciences, Government of India, Goa, IND; 2 Community Medicine, Teerthanker Mahaveer Medical College and Research Centre, Moradabad, IND

**Keywords:** occupational safety, mental health, preventive medicine, antarctica, public health

## Abstract

Public health in Antarctica is a pertinent issue that often gets overlooked. While the term 'public health' generally refers to the health of a larger community or the public, this concept is equally applicable to small, isolated populations, such as those residing in Antarctica. The principles of public health, including disease prevention, health promotion, and safety, are crucial for the well-being of those living and working in Antarctica. With the dramatic increase in tourist visits to Antarctica over the past decade, public health issues have become increasingly relevant and critical for the operation of base stations. In this article, we will discuss the need and relevance of public health for this growing community, the health issues they face due to extreme environmental conditions, and the measures to mitigate them.

## Editorial

Antarctica, often evoking mental imagery of floating icebergs and penguins, is commonly introduced to individuals through academic channels such as science or geography textbooks. Consequently, a prevailing perception emerges that penguins and seals, accompanied by a limited number of scientists and researchers, constitute the sole permanent inhabitants of the seventh continent. However, in the five decades since the establishment of the Antarctic Treaty in 1959, there has been a substantial escalation in the presence of scientific personnel. At present, nearly 30 countries maintain over 80 research bases across the Antarctic landscape, housing thousands of scientists [1]. This trend is anticipated to experience further acceleration in the coming years, indicative of a burgeoning scientific community dedicated to Antarctic exploration and research.

Though the term public health generally refers to the health of a community or the public at large, this concept can also be applied to small, isolated populations such as those living in Antarctica. Therefore, the principles of Public Health, including disease prevention, health promotion, and safety, remain relevant and critical for the well-being of those residing and working there. Yes, we are talking about the health and well-being of the expeditioners as well as tourists visiting this frozen continent. Even though the permanent number of residents in Antarctica is limited, the number of tourists visiting Antarctica has increased tremendously. More than 100,000 (1 Lakh) people visited Antarctica in 2022; hence, public health issues in Antarctica are extremely relevant and critical for the functioning of base stations [2]. Despite the small population size of the continent, the confined living spaces at most of the base stations create conditions conducive to the spread of communicable diseases. So, proper hygiene and preventive measures are crucial to avoid outbreaks. Individuals planning to travel/stay in Antarctica should ensure that they adhere to the vaccination against measles, mumps, and rubella (MMR), diphtheria/tetanus/pertussis (DTP), varicella, polio, and the yearly flu vaccine as per CDC guidelines [3]. Individuals from around the world coexist in confined spaces on cruise ships and polar icebreakers for weeks at a time, making it imperative that they are up-to-date on vaccinations. Additionally, quarantine measures should be followed before and after boarding the vessel. Travelers are also advised to receive COVID-19 vaccination. Food safety and health in Antarctica are of paramount importance and particularly challenging due to logistical hurdles in procurement and transportation, limited access to fresh produce, and extreme environmental conditions. Rigorous quality control, storage, and preservation methods are crucial to maintain nutritional adequacy during prolonged periods of darkness and extreme cold. Implementing strict food safety protocols, maintaining high hygiene standards, and providing adequate training are essential for preventing food-borne illnesses and promoting a positive food environment, which may contribute to a health-conscious Antarctic community. Mental health challenges in Antarctica are primarily due to the extreme cold conditions and isolation faced by research station personnel, particularly during the dark and cold nights of Antarctic winters, which can lead to feelings of loneliness, sadness, and polar night blues. Consequently, providing adequate mental health support services is crucial. These services should include stress mitigation strategies, counseling, and training to recognize the early signs of mental health issues. Additionally, the reintegration process upon returning home from Antarctica can be challenging, as individuals may struggle to adjust to the faster pace and complexity of human society. A comprehensive approach that combines on-site mental health support with post-return services is essential for monitoring, counseling, and facilitating a smooth transition back to normal life.

The occupational health and safety of station residents (scientists, support staff, and expedition team members), who form a small community in Antarctica, also present a set of unique challenges due to the extreme environment and isolation. They suffer from risks of cold-related injuries, slips, falls, musculoskeletal strains, and equipment-related accidents. The icy terrain and limited visibility during long winter nights heighten these risks. Limited access to medical facilities makes it even more challenging. To mitigate these risks, comprehensive training programs, ergonomic equipment, safety protocol, and regular drills are required to ensure that the expeditioners are well prepared to navigate the harsh environment of Antarctica [4]. This is essential for the scientific mission's success and the station's overall functioning.

Traveler's health is another issue. Tourists visiting Antarctica, even for short durations, face unique health challenges. Cold-related injuries and potential medical emergencies emphasize the need for well-equipped medical facilities and awareness of evacuation procedures. These aspects are crucial for a safe and enjoyable visit.

The concept of one health, which addresses health concerns at the human-animal-plant-environmental interface, also takes center stage in Antarctica. The impact of human presence is causing environmental disruptions that could pose new challenges to the continent's fragile ecosystem. These disruptions may allow diseases to pass to animals and vice versa. Recently (October 2023), the first case of Highly Pathogenic Avian Influenza (HPAI) was detected among the birds of the Antarctic region [5]. The World Health Organization (WHO) has raised concerns about the increasing presence of the H5N1 virus in mammals, emphasizing the potential for the virus to undergo adaptive changes that could increase its infectivity in humans. Human infrastructure also facilitates the introduction of invasive species, newer microbes into subglacial settings, and the use of anti-microbials and their impact on the delicate ecosystems of Antarctica.

Therefore, to effectively address these public health issues, Antarctic Programs from signatory countries should adhere to health and safety guidelines formulated by associations such as the Council of Managers of National Antarctic Programs (COMNAP) and the Scientific Committee on Antarctic Research (SCAR).
